# Finite element analysis of stress distribution on residual root structure in socket shield procedure following immediate dental implant placement: an in vitro study

**DOI:** 10.1186/s12903-024-04133-9

**Published:** 2024-03-21

**Authors:** Aniket Shinde, V.N.V Madhav, Ravinder S Saini, Vishwanath Gurumurthy, Rayan Ibrahim H Binduhayyim, Seyed Ali Mosaddad, Artak Heboyan

**Affiliations:** 1 Private Practice, Pune, India; 2 Department of Prosthodontics, YCMM and RDF’s Dental College & Hospital, 166/1, Vadgaon Gupta, Opp MIDC, Ahmednagar, Maharashtra 414003 India; 3 Department of Dental Technology, COAMS, King Khalid University, Abha, Saudi Arabia; 4 Department of Research Analytics, Saveetha Institute of Medical and Technical Sciences, Saveetha Dental College and Hospitals, Saveetha University, Chennai, India; 5 Student Research Committee, School of Dentistry, Shiraz University of Medical Sciences, Qasr-e-Dasht Street, Shiraz, Iran; 6 Department of Prosthodontics, Faculty of Stomatology, Yerevan State Medical University after Mkhitar Heratsi, Str. Koryun 2, Yerevan, 0025 Armenia; 7 Department of Prosthodontics, School of Dentistry, Tehran University of Medical Sciences, Tehran, Iran

**Keywords:** Dental implants, Finite element, Residual root structure, Stress, Socket shield technique

## Abstract

**Background:**

The success of implants in the socket shield technique relies on stress experienced by root fragments within the socket. Although there is no consensus on optimal root fragment thickness, varying thicknesses and dynamic implant placement induce stress in various directions and degrees. This study aimed to assess biomechanical response and stress distribution across different root fragment thicknesses in the socket shield procedure.

**Methods:**

This in vitro study was conducted to assess and compare the stress distribution on residual root structures of varying thicknesses positioned within the labial aspect of the maxillary incisor socket during immediate implant placement of standard dimensions. The procedure involved applying an insertional torque of 40 Ncm, and the analysis was conducted using finite element analysis software.

**Results:**

Utilizing the Numerical Technique with Abaqus software for explicit dynamics, von Mises stress and principal strain were analyzed on the root structure and bone under nonlinear contact conditions during implant torque application. For Model A, a loading torque of 40 Ncm was applied vertically on the implant, along with a horizontal force of 20 N on the root structure and bone. Results indicated maximum stress of 12.68 MPa on the root structure with a thickness of 0.5 mm and 5.61 MPa on the bone, with principal strains of 6.82E-03 and 4.10E-03, respectively. In Model B, with a root structure thickness of 1.0 mm, the maximum stress increased to 19.70 MPa, while the bone stress rose to 9.51 MPa, with principal strains of 1.03E-02 and 6.09E-03. Model C, with a root structure thickness of 1.5 mm, exhibited a maximum stress of 21.58 MPa on the root and 10.12 MPa on the bone, with principal strains of 1.16E-02 and 6.10E-03. Lastly, in Model D, with a root structure thickness of 2.0 mm, the maximum stress on the root structure and bone escalated to 28.74 MPa and 11.38 MPa, respectively, with principal strains of 1.55E-02 and 8.31E-03.

**Conclusions:**

As the thickness of the shield increases (ranging from 0.5 to 2 mm) in socket shield procedures with immediate implant placement, both stress on the root fragment and bone and principal micro-strain escalate. However, employing a shield thickness within the range of 0.5 to 2 mm does not lead to any adverse stress generation on the residual root fragment. However, for enhanced safety, it is recommended to restrict the maximum diameter and extension of the root fragment to 1.5 mm when considering implant sizes and socket diameter for the socket shield technique.

## Background

When a tooth is lost, dental implants are often used to replace it. Traditional implants are usually used only when the extraction site has a healthy, edentulous alveolar ridge. This requires a healing period of approximately three to six months, during which the bone undergoes remodeling. However, over time, the alveolar bone may experience significant resorption, which can result in unfavorable implant positioning and aesthetic concerns, especially in the anterior maxilla. To address these challenges and meet patient preferences for shorter treatment times and fewer surgeries, immediate implant placement has been introduced as a safe and effective alternative [1].

In prosthetic rehabilitation, a significant aim is to achieve and sustain a harmonious balance between the pink (soft tissue) and white (tooth) zones, particularly in areas where aesthetics are crucial. However, it has been observed that performing gentle and non-traumatic tooth extractions with immediate implant placement can lead to undesirable outcomes. This includes loss of the buccal bone, both vertically and horizontally, as well as flattening of the interproximal bony scallop. These complications can further complicate the rehabilitation process [2].

Loss of ridge volume and alterations to ridge shape can occur after tooth extraction, sometimes to the extent of 3.8 mm in the horizontal and 1.24 mm in the vertical direction. Buccal alveolar ridge resorption is more pronounced than lingual resorption. Loss of the periodontal ligament and any subsequent trauma, especially to the buccal bone plate, account for the major changes observed after tooth extraction. Therefore, it is reasonable to infer that root retention affects resorption. Several solutions, including the socket shield method, have been proposed to address this problem [3].

In 2010, Hurzeler et al. introduced a groundbreaking technique known as the socket-shield technique. Its purpose was to prevent the loss of the facial ridge that often occurs after tooth extraction and immediate implant placement. What makes this method unique is its ability to preserve a portion of the periodontal ligament adjacent to the root fragment, minimizing the aesthetic impact of bone remodeling. Follow-up X-ray examinations conducted at 6-month intervals revealed that the conventional technique resulted in a considerable 5 mm loss of bone tissue. In contrast, the socket shield approach showed a significantly lower loss of only 0.8 mm. These outcomes have had a direct and positive influence on the volume of soft tissue, leading to a more natural appearance, as confirmed by both patients and clinicians. Socket shielding has been shown to be an effective method for preventing bone loss and, hence, the requirement for soft-tissue grafts. Socket shields should have a thickness of at least 1.5 mm and preferably 2 mm, as stated in the referenced literature. A lack of research, however, has examined the effect of varying root fragment thicknesses while using this method. Therefore, the stress levels on different root thicknesses in conjunction with the socket shield approach and rapid dental implant insertion were evaluated using finite element analysis (FEA). In addition, ideal torque values were used in this investigation for reliability and thoroughness [4].

In many cases, it is challenging to maintain the desired distance between the root fragment and implant during torque application. This can lead to the generation of stress in the root fragments. Without the use of torque measuring devices, an average surgeon may achieve an average insertion torque of approximately 63.26 + 6.8 Ncm. However, it is important to note that using an insertion torque of 70 Ncm or higher during the placement of a wide-diameter implant immediately after extraction can significantly increase the stress on the crestal bone. The development of a modified surgical protocol is recommended to reduce the risk of early implant failure in extraction sockets. This protocol involves the use of a moderate insertion torque value ranging from 32 to 50 Ncm. By following this protocol, the aim is to minimize stress on the root fragment and improve the success rate of immediate implant placement [5].

The finite element method is a valuable tool that can be used to evaluate and simulate the complex biomechanical aspects of surgical situations. It provides a comprehensive and reliable means of analyzing the mechanical properties of complex structures. FEA has emerged as a significant and promising tool for biomechanical analysis in the field of biological research. FEA has proven to be particularly useful in the field of implantology. It allows researchers to study stress patterns in different implant components and peri-implant bones, assess the biomechanical properties of implants, and predict their success under clinical conditions. FEA has many advantages over traditional research methods that use physical models. However, research has different views on the ideal thickness of root fragments for use in socket shield techniques and how stress is distributed among root fragments of varied thicknesses. Socket shield immediate dental implants are becoming increasingly common, and this study aimed to determine how biomechanical response and stress distribution vary among root segments of varying thicknesses in such operations [6].

## Methods

The study was conducted at the Department of Prosthodontics, YCMM, and RDF’s Dental College in Ahmednagar, Maharashtra, India. The purpose of this in vitro investigation was to assess and contrast the distribution of stress on residual root structures of different thicknesses when an immediate implant of standard dimensions was placed in the labial aspect of the socket of the maxillary incisor. The study employed finite element analysis software to analyze the stress distribution with an insertional torque of 40 Ncm.

### Materials

**Fig. 1:**
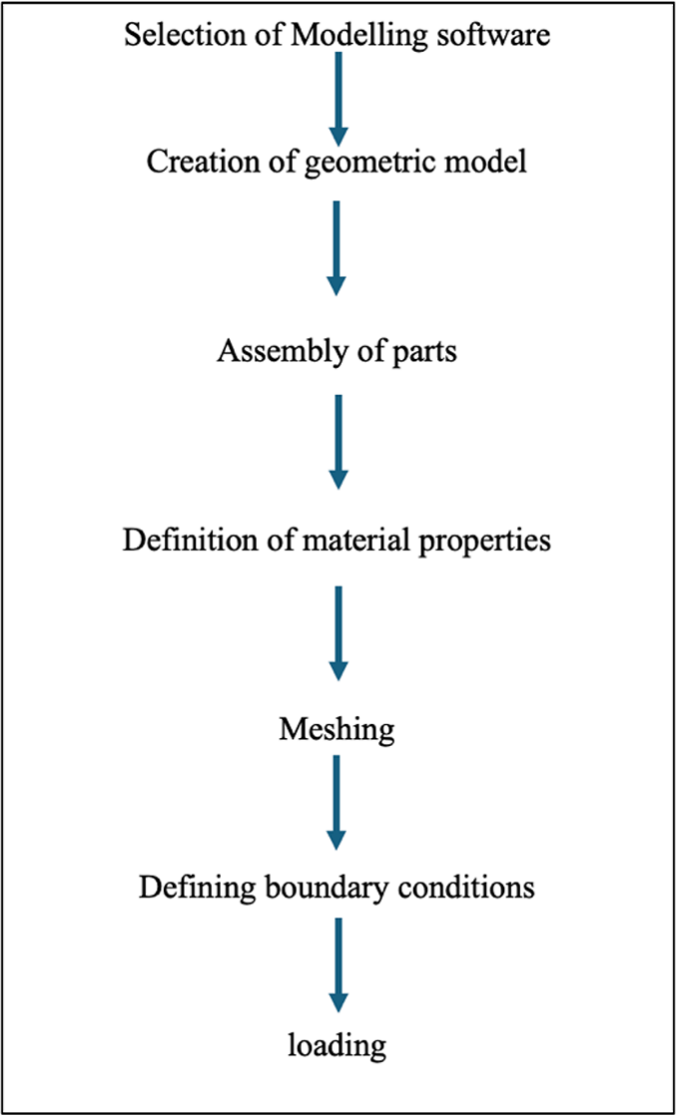
Flow chart of various steps employed in the current study

1. Adin implant of dimensions 4 × 11.5 mm (Fig. 1) (Adin WP closeFitTM)
2. Solidworks 2019 CAD design software (Dassault Systèmes SOLIDWORKS Corp)
3. ABAQUS 2021 finite element analysis software (ABAQUS 2021- Dassault Systèmes)
4. Hypermesh 2018 finite element meshing software (Altair HyperMesh)

### Methodology (schematic diagram, Fig. 1)

Steps in Simulation:

1. Modelling software
2. Creation of a geometric model
3. Assembly of parts.
4. Definition of material properties
5. Meshing of parts by assigning elements, element properties, and sections assigned for the same
6. Type of analysis and Defining boundary conditions and loading
7. Analysis of results

#### Modeling software

The approach taken in this study involved creating a geometric model of the jawbone using Solidworks software (2019). The bone was modeled in two states: cortical and cancellous. A geometric model of the implant set and its cover was constructed using SolidWorks software and inserted into the bone.

The properties and conditions for the boundary and force were determined after importing the geometric models into the ABAQUS analytical software (2021). The model was then evaluated and examined after meshing to ensure its accuracy. FEA was conducted with mechanical loads applied to the model.

To improve the precision of the simulation results, the element sizes were adjusted to ensure convergence. The outcomes of this research were compared with the results from reputable articles to validate the findings. In this study, it was assumed that there was perfect contact between the jaw and implant, with no slippage. This assumption was considered during the static analysis conducted in the study.

#### Creation of geometric model

**Fig. 2:**
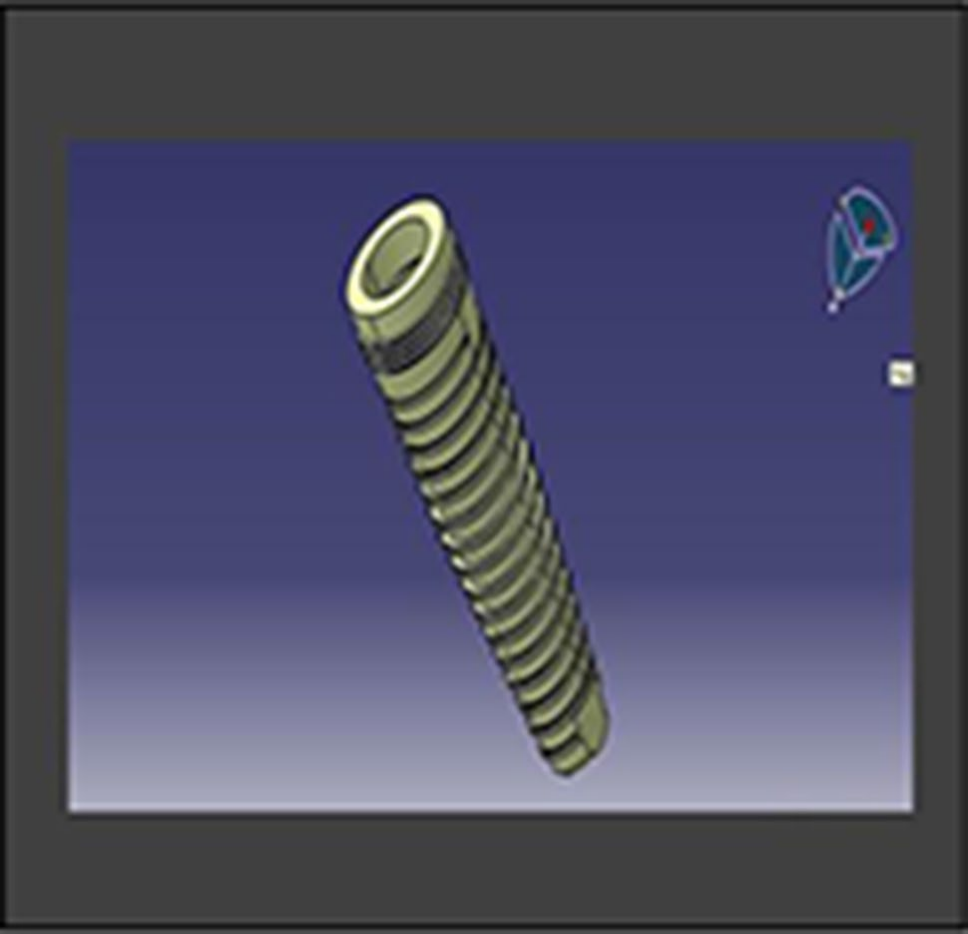
Implant CAD model of Dimensions 4 × 11.5 mm

A 3D implant model (Adin WP closeFitTM) was utilized to examine the stress distribution caused by various treatments; this model had a broad platform connection and measured 4 mm × 11.5 mm (Fig. 2). An optical profile measuring machine was employed for this purpose.

**Fig. 3:**
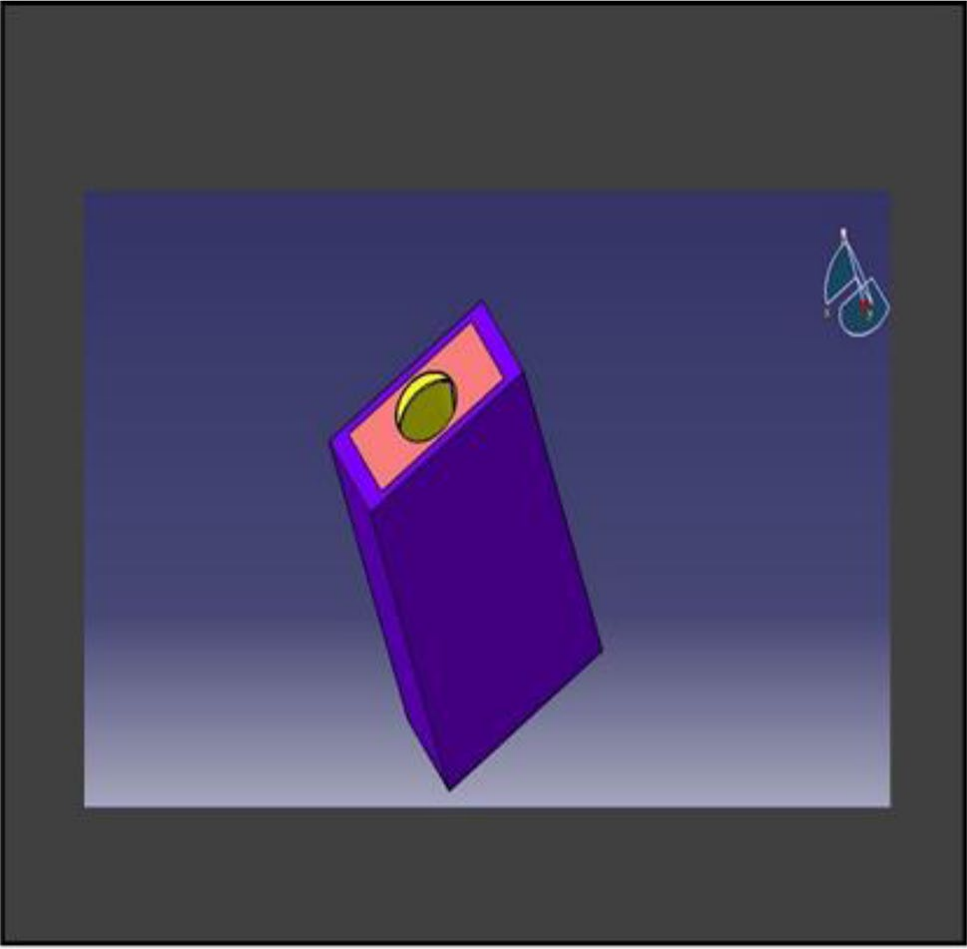
3D CAD Model of extraction socket in Anterior Maxilla

**Fig. 4:**
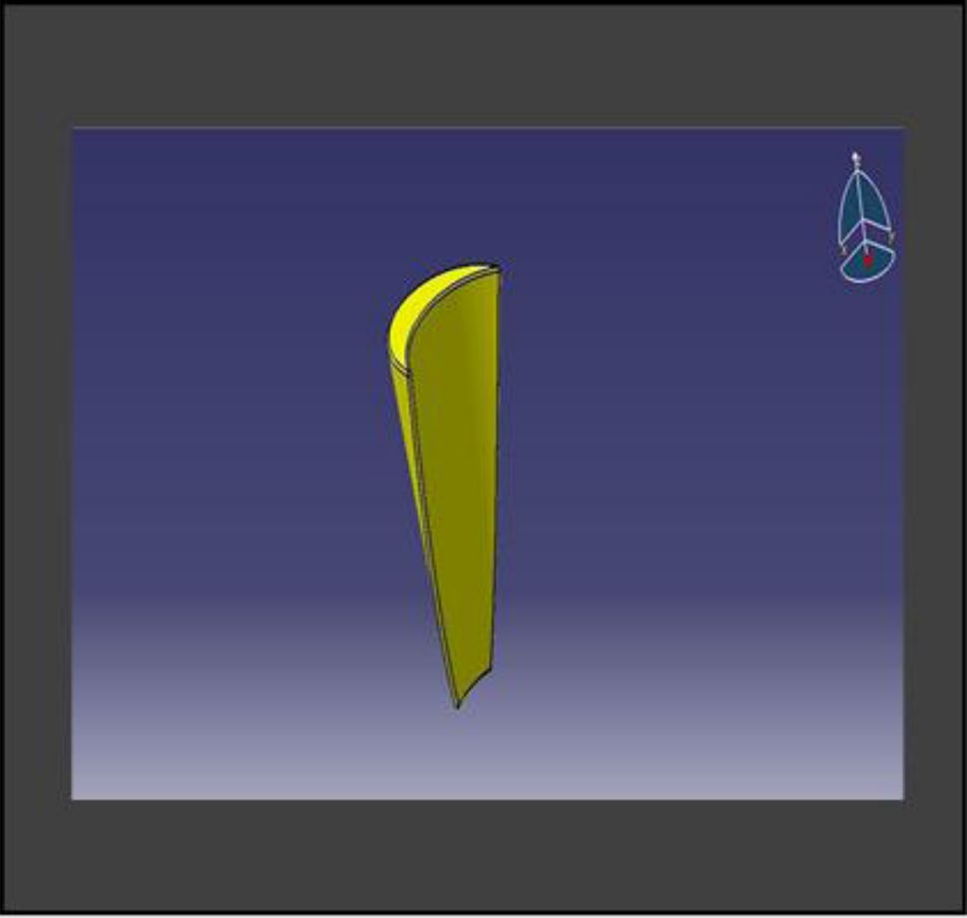
3D CAD model of root structure used for socket shield

The model was transferred into CAD software (Solidworks 2019). Using this software, a model of an extraction socket in the anterior maxilla was created, including both cortical bone and cancellous bone (Fig. 3). Additionally, models of the root structures with dimensions of 0.5 × 11 mm, 1 × 11 mm, 1.5 × 11 mm, and 2 × 11 mm (Fig. 4) were generated. These models were divided into four groups to simulate different clinical situations. Each group underwent a specific surgical procedure immediately after implant installation, incorporating socket shields of varying thicknesses.

#### Assembly of parts

**Fig. 5:**
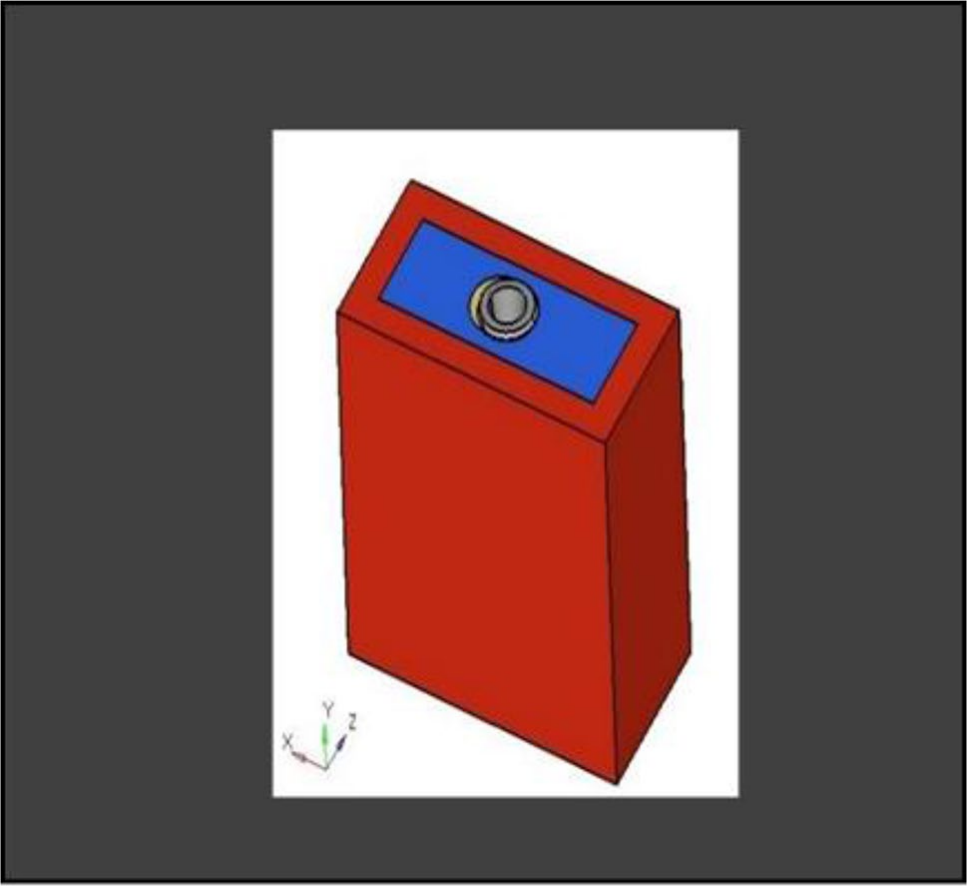
Assembly of parts

Different pieces of the model, including the implant, were positioned adjacent to each other, and their relative positions were defined for this reason. Figure 5 depicts the model parts being combined.

### Defining material properties

The mechanical properties of the materials used in this study were obtained from various sources in the literature. These properties are summarized in Table 1, with references to the respective studies (Rees et al. 1994; Madfa et al. 2014; Tribst et al. 2017; Monteiro et al. 2018) [7].

**Table 1:** Mechanical properties of the materials and structures used in this study

Material/Structure	Elastic modulus (GPa)	Poisson’s ratio	Reference
Titanium	110	0.32	Tribst et al. 2017
Cortical bone	13.7	0.30	Madfa et al. 2014
Cancellous bone	1.37	0.30	Madfa et al. 2014
Root dentin	18.6	0.31	Rees et al. 1994

#### Meshing

**Fig. 6:**
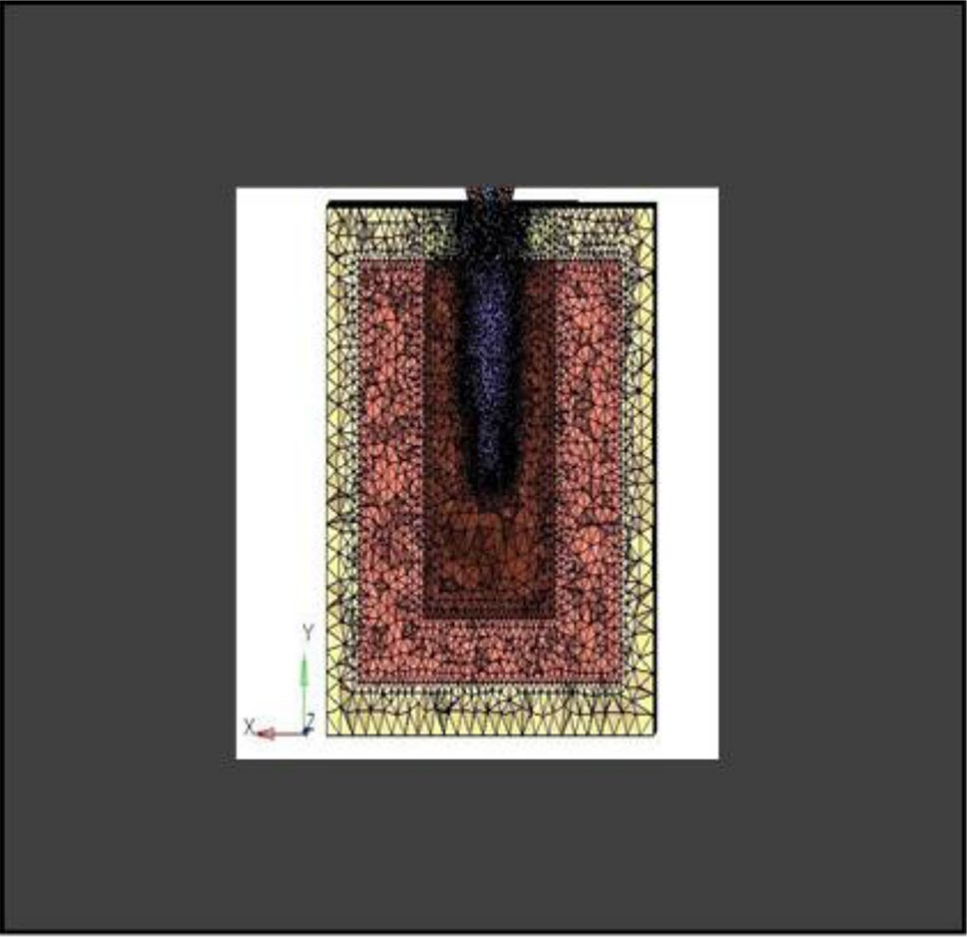
Meshing Convergence at the interface

Partial Differential Equations (PDEs) are commonly used to solve various physical phenomena. However, solving real-world problems is challenging owing to the infinite degrees of freedom (DOF) associated with continuous objects. However, manual calculations are not feasible in such cases. Therefore, Finite Element Analysis (FEA) is employed, where a mesh is created to discretize the domain into a finite number of elements, allowing for the calculation of the solution. The data were interpolated across the entire domain. Accurate results from FEA models rely heavily on meshing because the elements in the mesh must consider multiple factors to accurately represent the stress gradients. Generally, a smaller mesh size provides a more precise solution because the design is better sampled across physical domains. In this study, the CAD model was imported into the Hypermesh software (version 18.0)(Fig. 6) for complete meshing of the socket shield assembly. Tetrahedral elements with a size of 0.1 mm were utilized. Table 2 provides details of the number of nodes and elements used in this study.

**Table 2:** Finite model nodes and elements with different shield thickness

Shield thickness (mm)	No. of elements	No. of nodes
0.5	1,00,223	1,48,535
1.0	1,12,635	1,53,438
1.5	1,23,127	1,62,923
2.0	1,34,692	1,71,241

### Type of analysis and defining boundary conditions and loading

After the development of each model, numerical simulations were conducted using the ABAQUS 2021 analytical program. Simulations were conducted using the finite element approach. A mesh convergence test was performed to ascertain the precision of the outcomes. The experiment entailed a systematic process of progressively transitioning and meticulously refining the mesh to establish the ideal quantity of elements and nodes necessary for each geometric configuration.

**Fig. 7:**
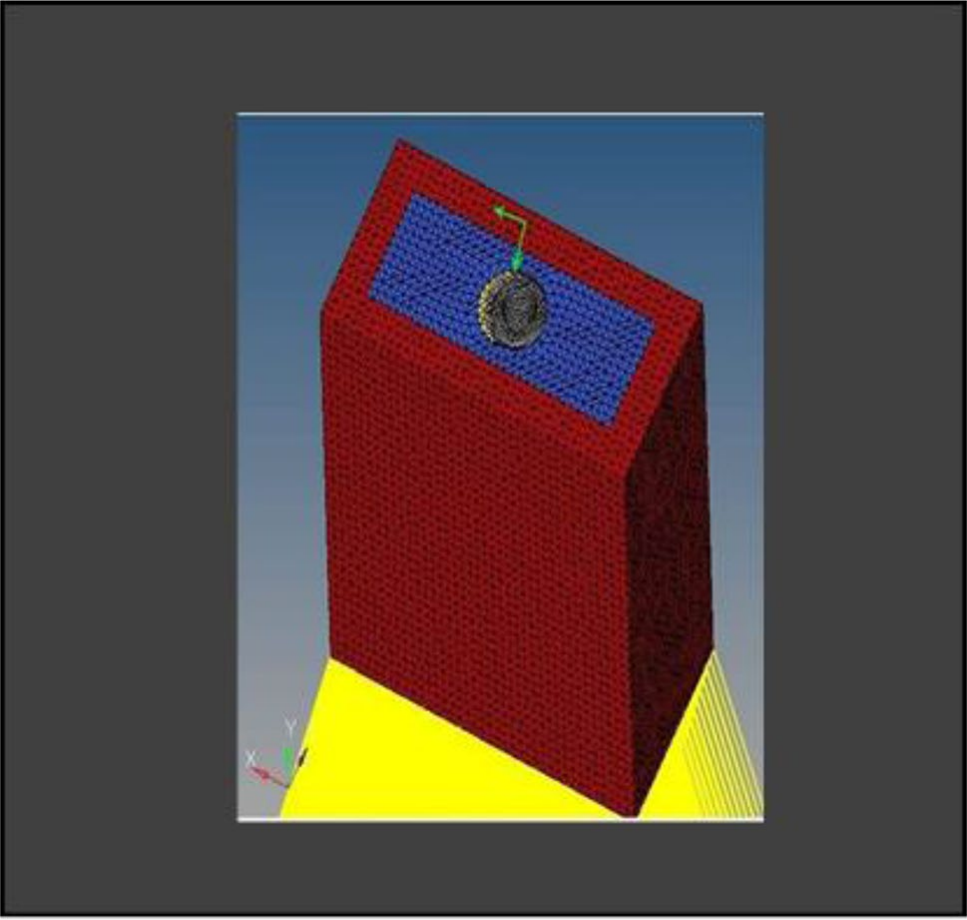
Loading Constraints

Several standardizations were implemented to ensure consistency in the analyses. All materials were assumed to be homogeneous, elastic, and isotropic, with similar structures having identical contact properties. A perfect bond was assumed between the root and bone structures. The selection of the reference point for loading was carefully considered. In this study, a continuous implantation process with a constant torque was simulated. Specifically, a vertical torque of 40 N · cm was applied to the top of the implant, whereas a horizontal force of 20 N (equivalent to an insertion torque of 40 Ncm) was applied from the inner side of the root structure. The bottom one-third of the cortical bone was subjected to constraints in all directions, as seen in (Fig. 7). The complicated challenge of modeling the interaction between the implant, root structure, and bone during static modeling of the implantation process necessitates the establishment of suitable contact conditions. In the present investigation, the concept of contact was operationalized through the use of “surface-to-surface” discretization inside the ABAQUS software. This particular approach was chosen because of its superior ability to provide more precise stress and pressure outcomes when compared to the alternative node-to-surface discretization method. The implementation of surface-to-surface contact involves the establishment of constraint enforcement techniques that do not necessitate the alignment of meshes, such as the use of node-to-node contact. In the ABAQUS software, conditional restrictions are applied to each surface to accurately mimic the contact situations. In addition, the contact pairs were characterized by nonlinear contact interaction features, with a friction coefficient of 0.30, as recommended by Grant et al. (2007) [8], for the loading process.

**Fig. 8:**
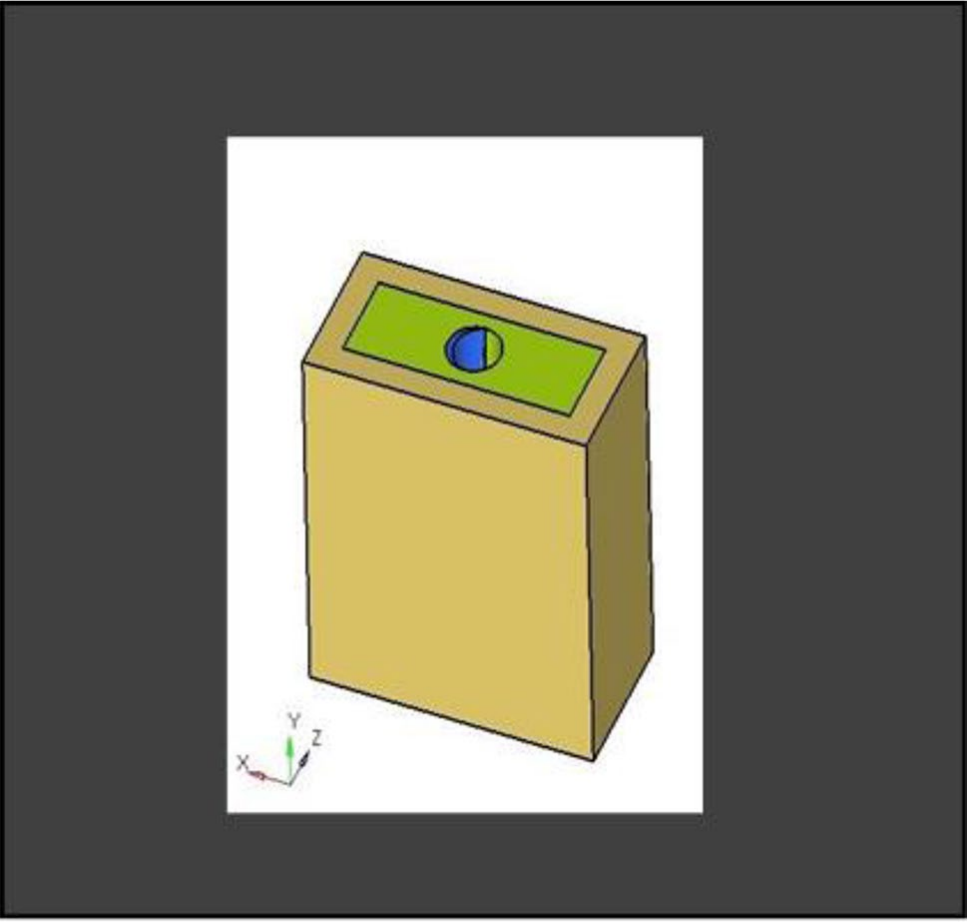
3D CAD Model of Extraction socket with socket shield of thickness 0.5 mm

**Fig. 9:**
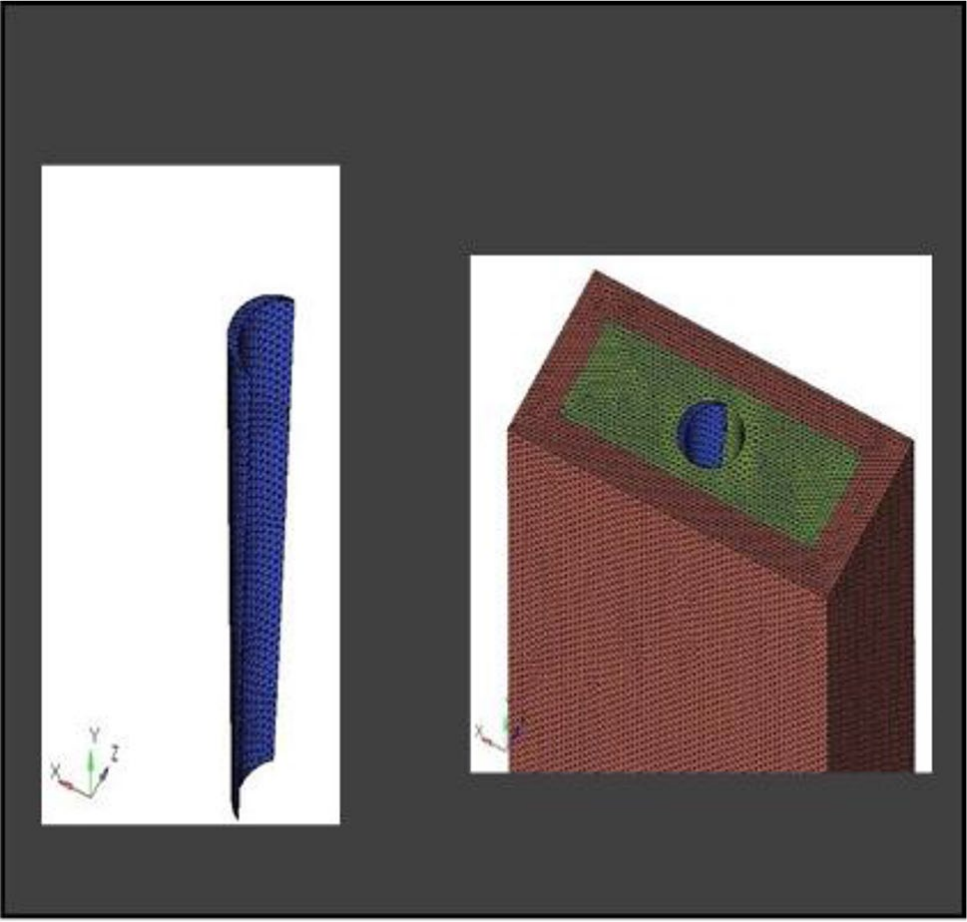
Meshing of model of extraction socket and socket shield of thickness 0.5 mm

**Fig. 10:**
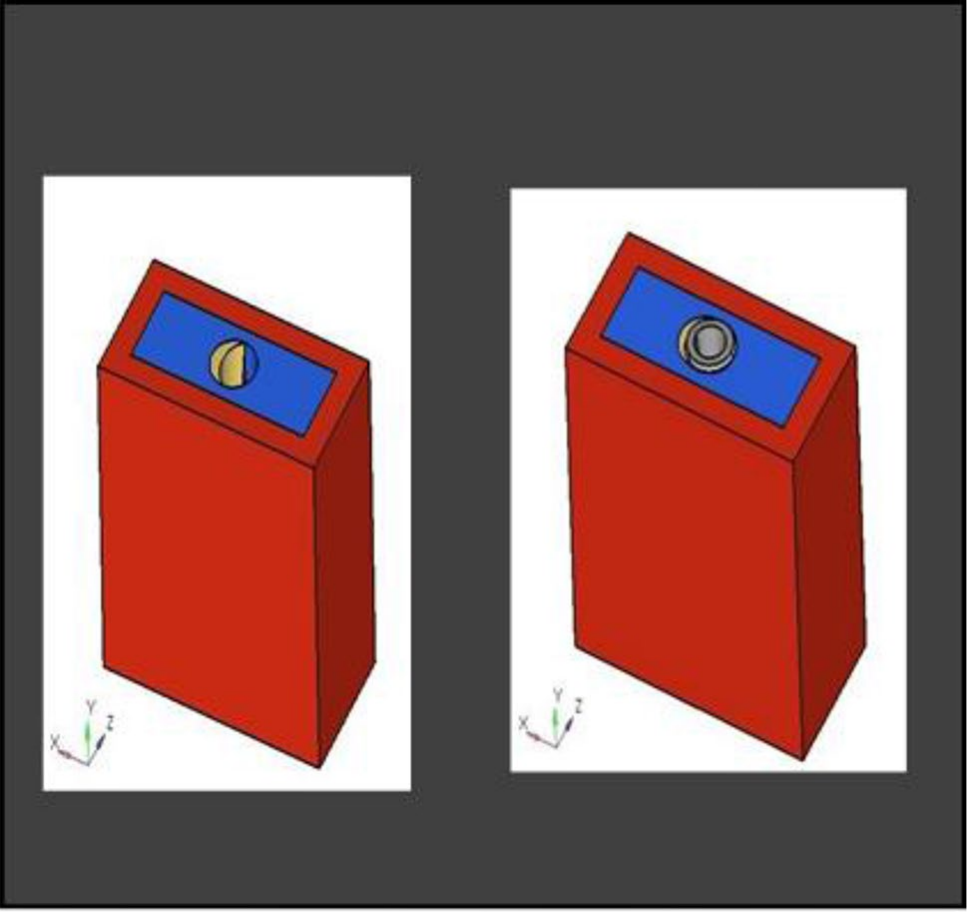
3D CAD model of extraction socket with socket shield of thickness 1 mm with implant

**Fig. 11:**
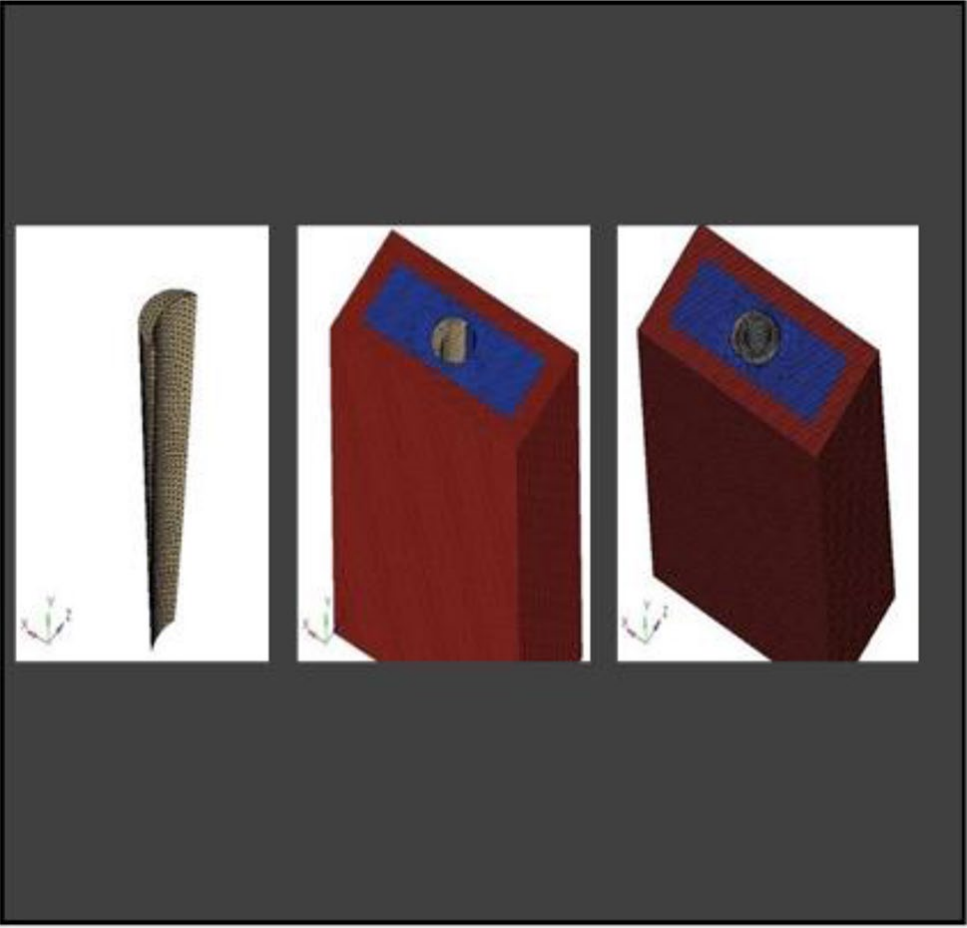
Meshing of extraction socket model, socket shield of thickness 1 mm and implant

**Fig. 12:**
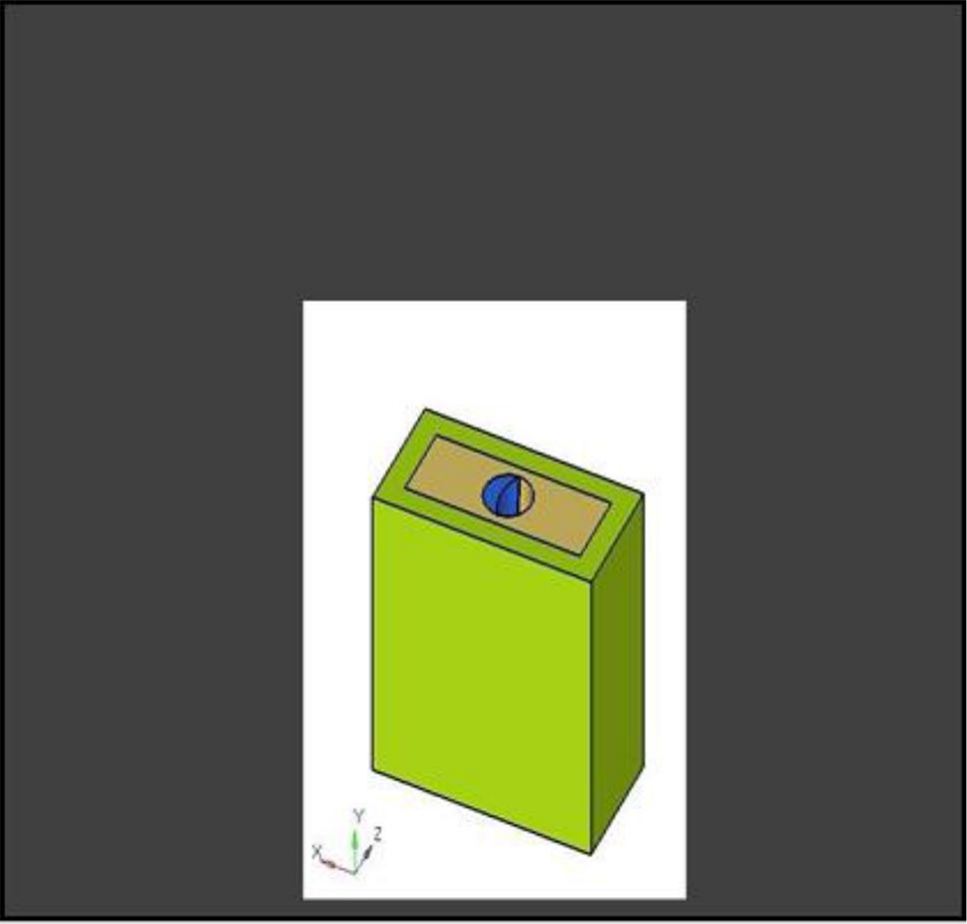
3D CAD Model of extraction socket with socket shield of thickness 1.5 mm with implant

**Fig. 13:**
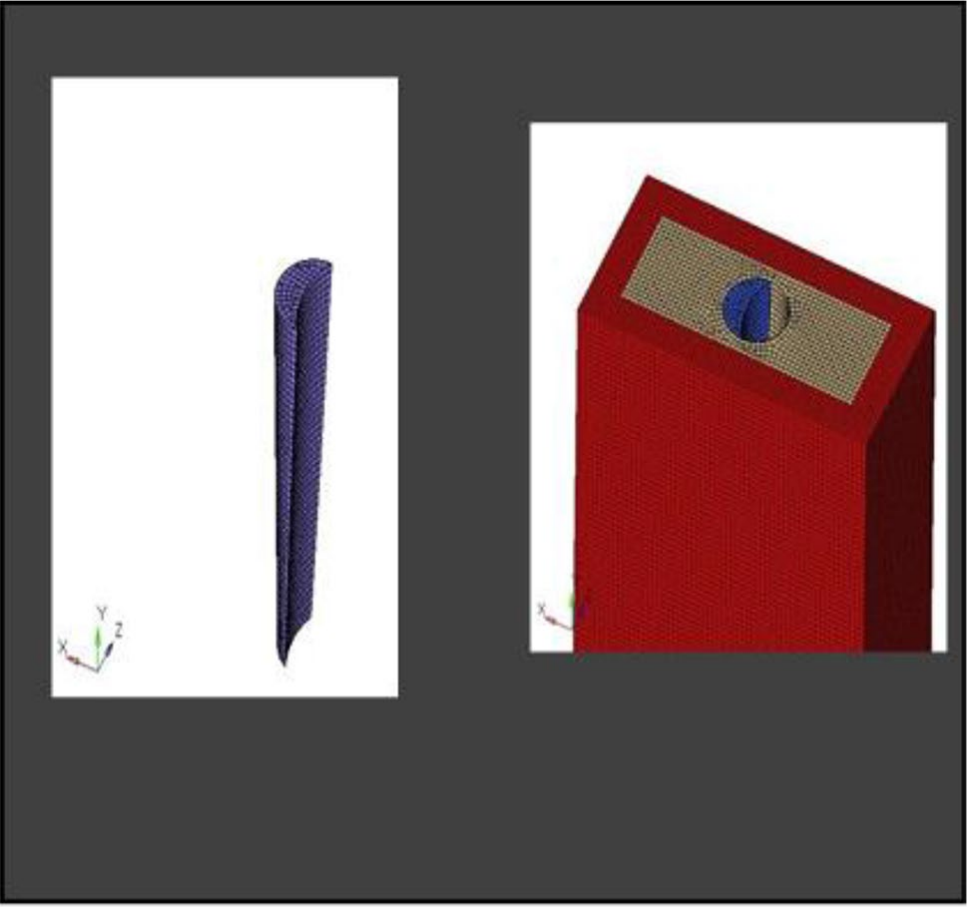
Meshing of extraction socket model and socket shield of thickness 1.5 mm

**Fig. 14:**
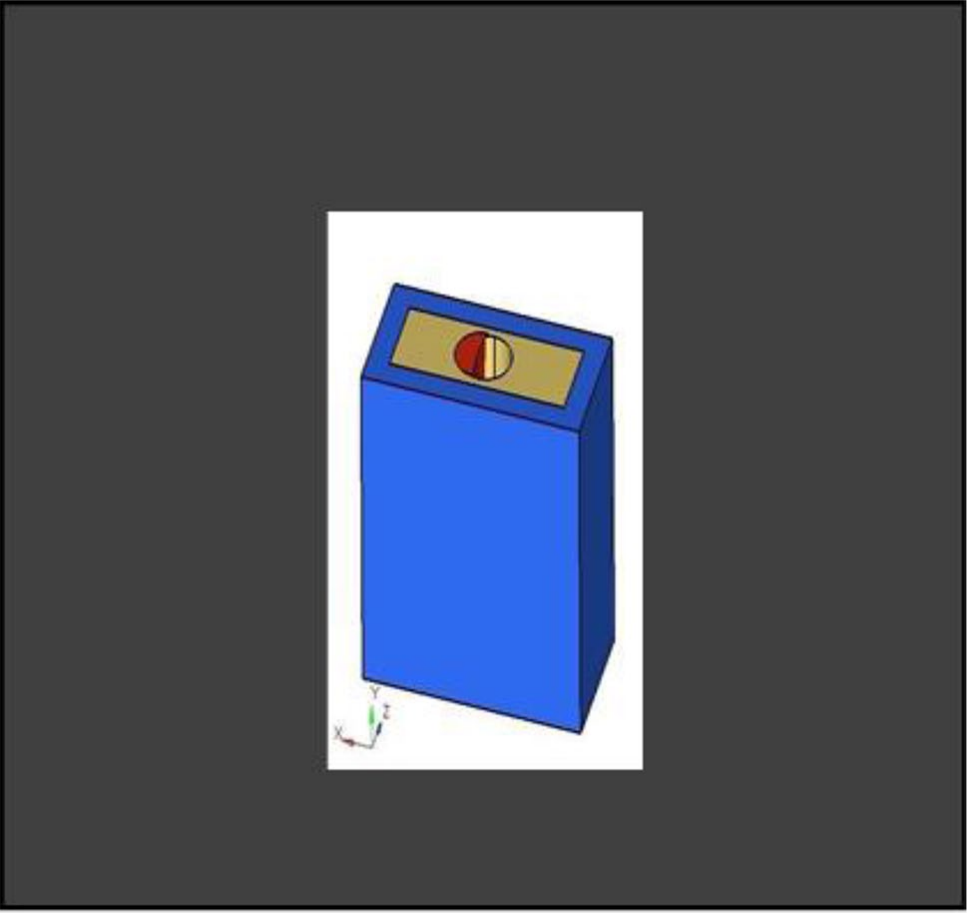
3D CAD model of extraction socket with socket shield of thickness 2 mm

**Fig. 15:**
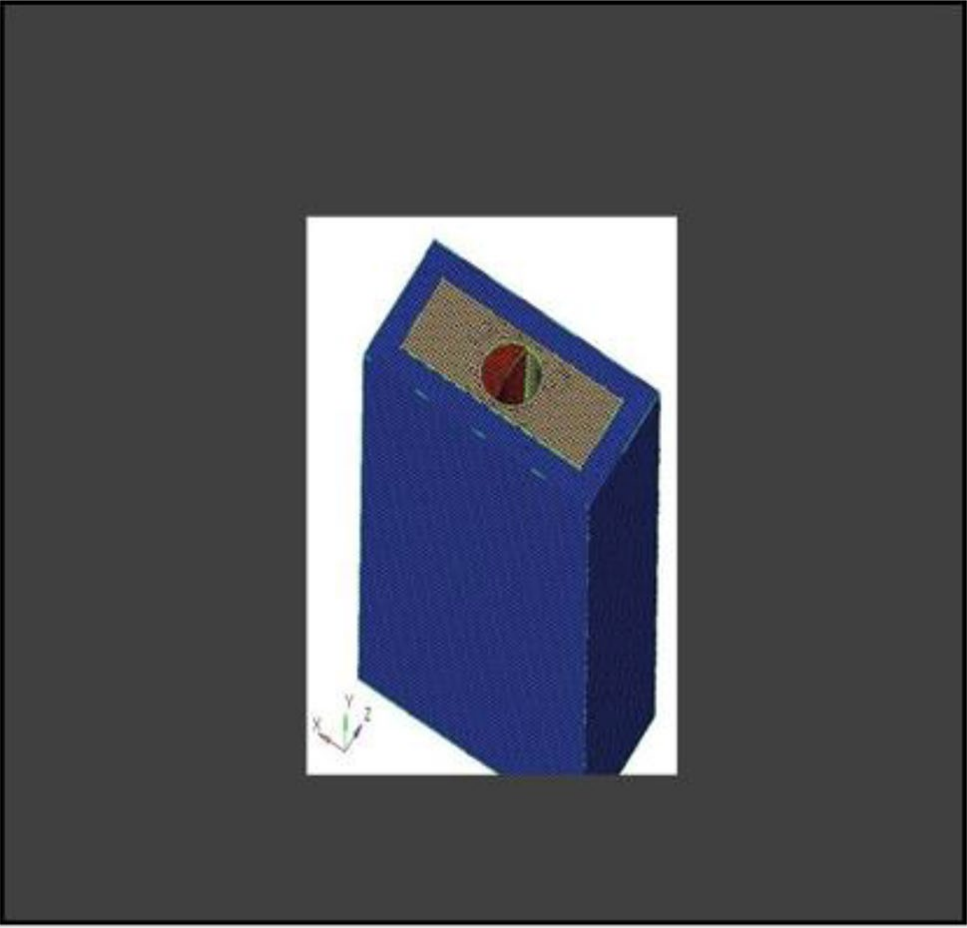
Meshing of extraction socket with socket shield of thickness 2 mm

Different groups were created in this study with reference to the socket shield thickness as follows:

- **Model A:** implant (4 × 11.5), model of the socket in the anterior maxilla and model of root structure (0.5 × 11) (Fig. 8) and meshed model of the same geometric dimensions (Fig. 9)
- **Model B:** implant (4 × 11.5), model of the socket in the anterior maxilla and model of root structure (1 × 11) (Fig. 10) and meshed model of the same geometric dimensions (Fig. 11)
- **Model C:** implant (4 × 11.5), model of the socket in the anterior maxilla and model of root structure (1.5 × 11) (Fig. 12) and meshed model of the same geometric dimensions (Fig. 13)
- **Model D:** implant (4 × 11.5), model of the socket in the anterior maxilla and model of root structure (2 × 11) (Fig. 14) and meshed model of the same geometric dimensions (Fig. 15)

## Results

This study aimed to evaluate the stress induced on root fragments measuring 2 mm in thickness when subjected to a torque of 40 Ncm. The analysis aimed to visualize the stress distribution on the bone and socket shield using color coding, with blue indicating minimal stress and red indicating maximum stress. To achieve this, Abaqus software was employed for explicit dynamic analysis, allowing for the determination of von Mises stress and principal strain on both the root structure and bone. Nonlinear contact conditions were considered when applying torque to the implant.

### Loading results of model A

**Fig. 16:**
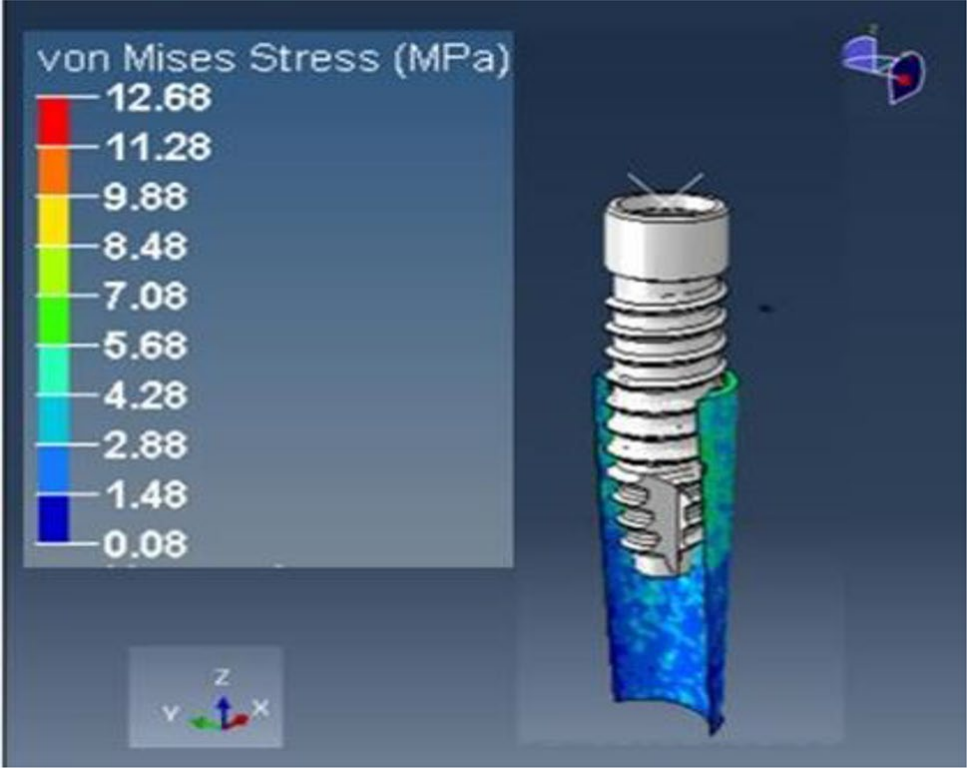
Stress on the shield of thickness 0.5 mm

**Fig. 17:**
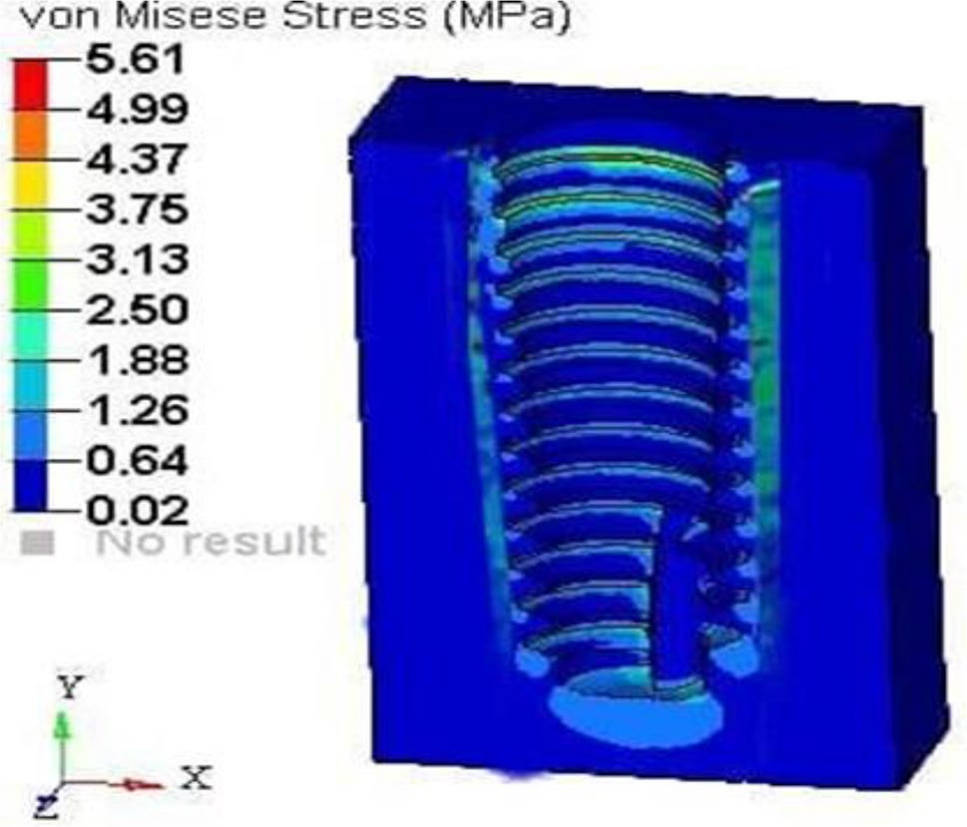
Stress on the bone of SS of thickness 0.5 mm

A vertical loading torque of 40 Ncm was exerted on the implant, whereas a horizontal force of 20 N was applied to the root structure and bone. The analysis showed maximum stress on the 0.5 mm thick root structure at 12.68 MPa (Fig. 16) and on the bone at 5.61 MPa (Fig. 17), with principal strains of 6.82E-03 and 4.10E-03, respectively.

### Loading results of model B

**Fig. 18:**
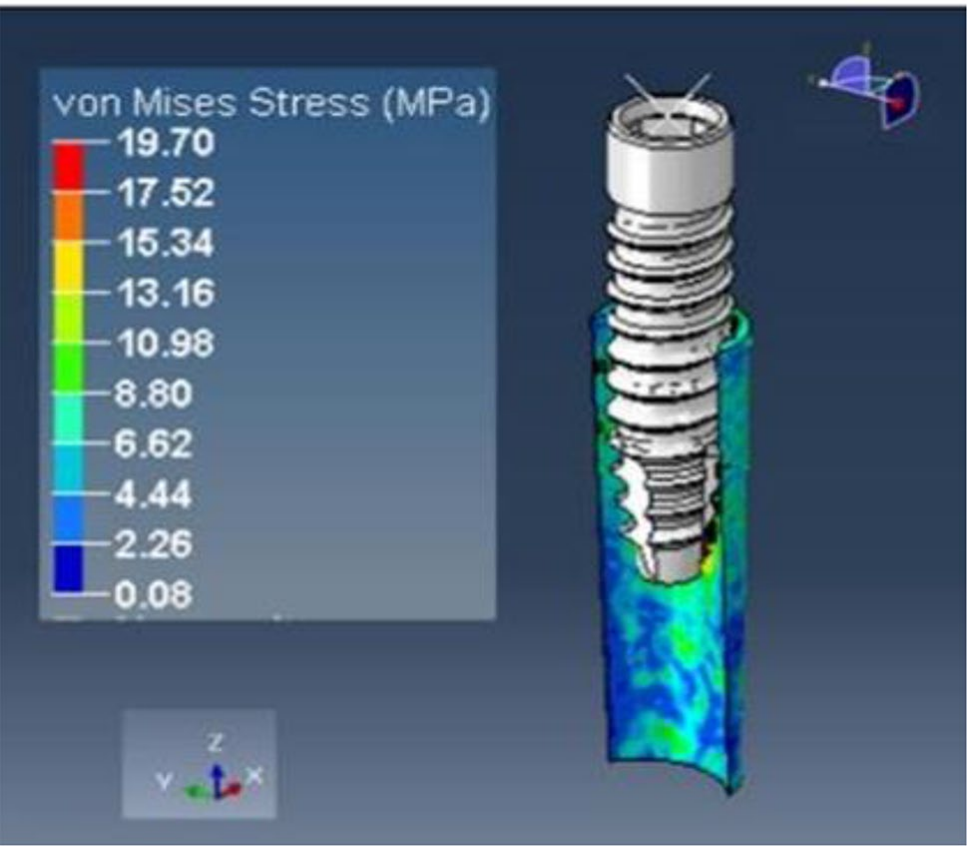
Stress on the shield of thickness 1.0 mm

**Fig. 19:**
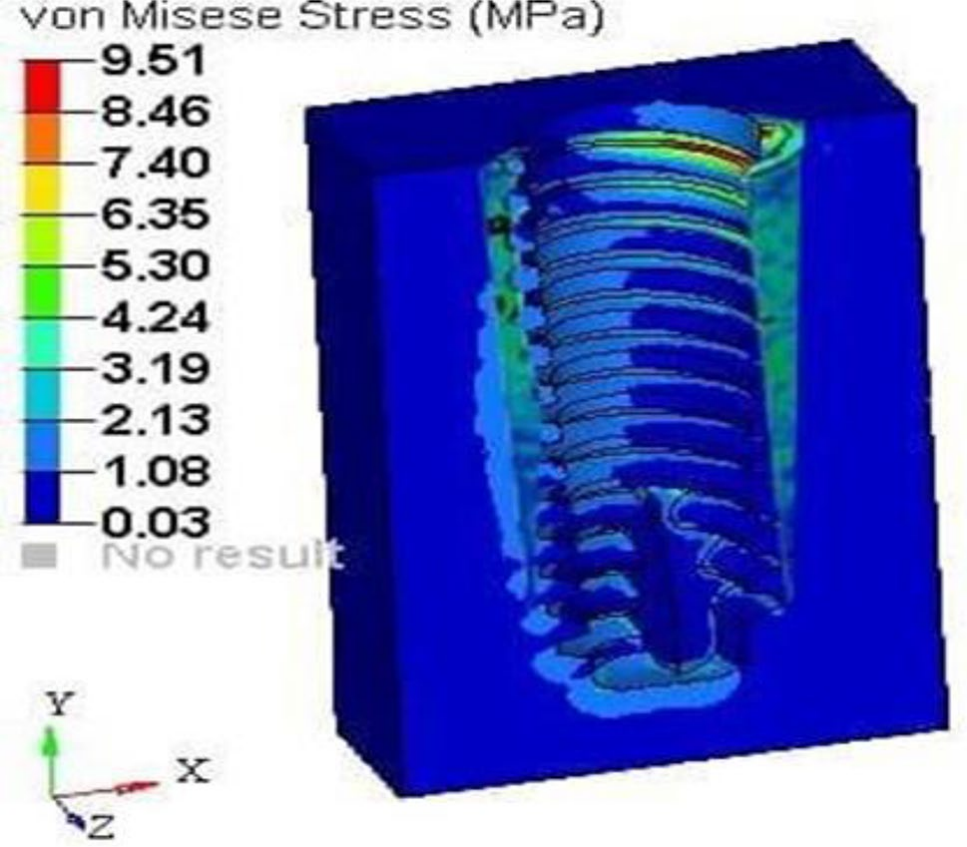
Stress on the bone of SS of thickness 1 mm

The implant was subjected to a vertical loading torque of 40 Ncm, whereas the root structure and bone were loaded with a horizontal force of 20 N. It showed maximum stress on the root structure of thickness 1.0 mm of 19.70 (Fig. 18) Mpa and on the bone of 9.51 (Fig. 19) Mpa and principal strains of 1.03E-02 and 6.09E-03, respectively.

#### Loading results of model C

**Fig. 20:**
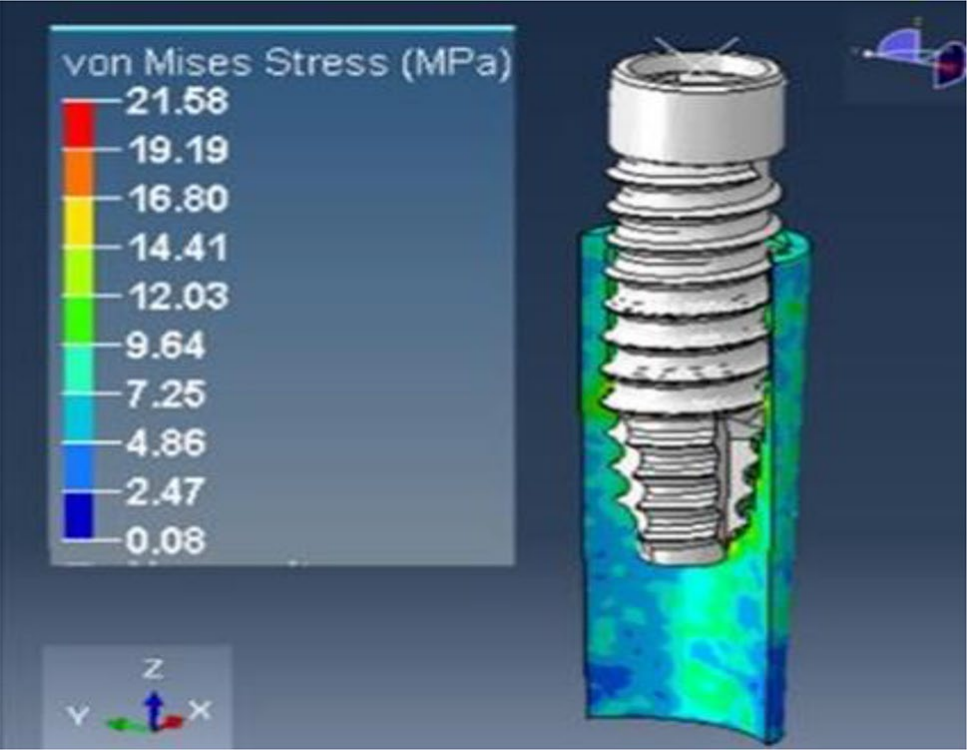
Stress on the shield of thickness 1.5 mm

**Fig. 21:**
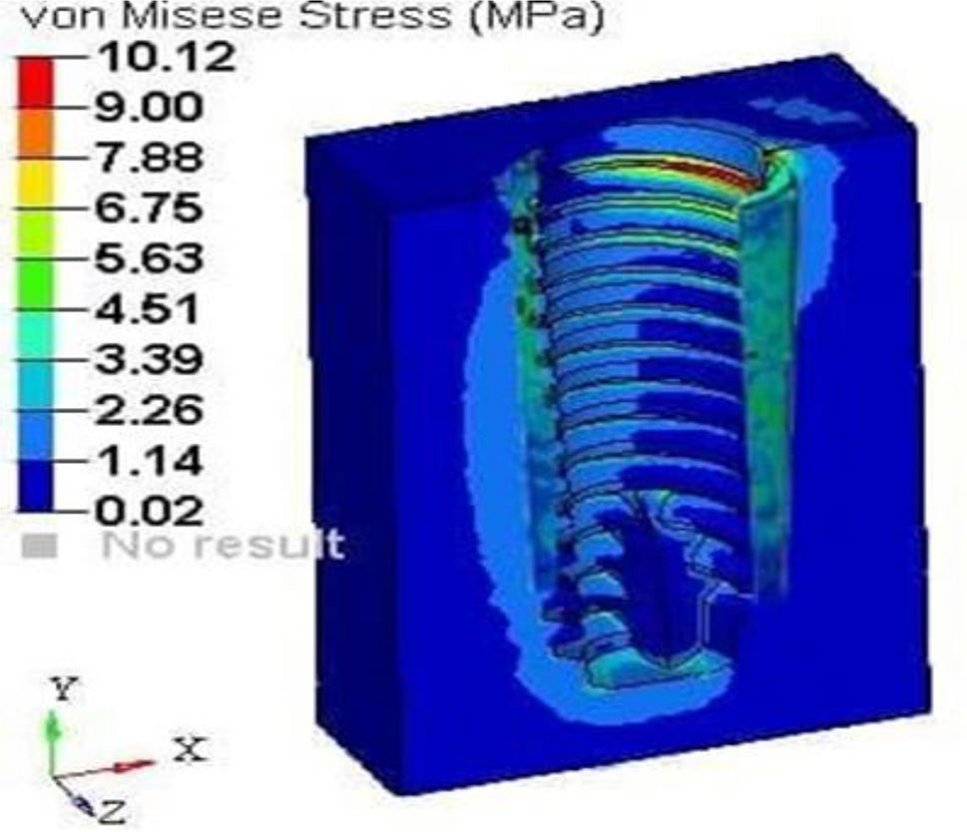
Stress on the bone of SS of thickness 1.5 mm

A loading torque of 40 Ncm was applied vertically on the implant, and a force of 20 N was applied horizontally on the root structure and bone. The analysis revealed peak stress on the root structure, measuring 1.5 mm in thickness, at 21.58 MPa (Fig. 20), and on the bone at 10.12 MPa (Fig. 21), with principal strain values of 1.16E-02 and 6.10E-03, respectively.

### Loading results of model D

**Fig. 22:**
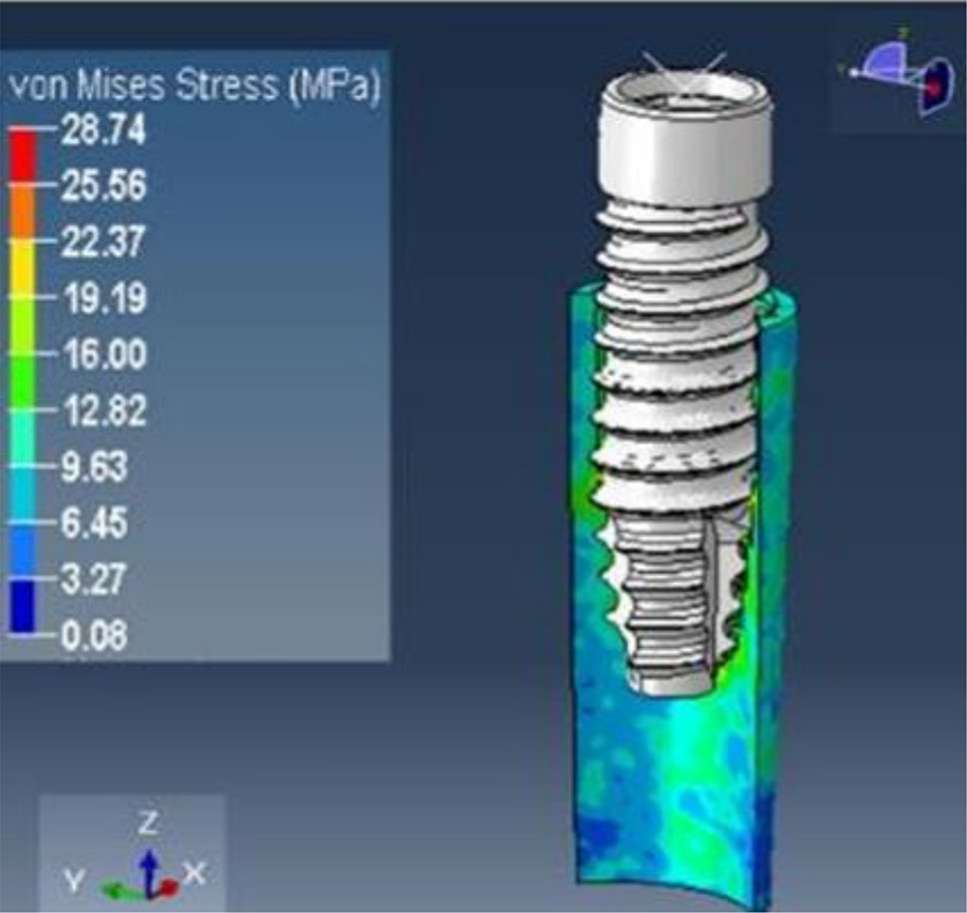
Stress on the Shield of thickness 2.0 mm

**Fig. 23:**
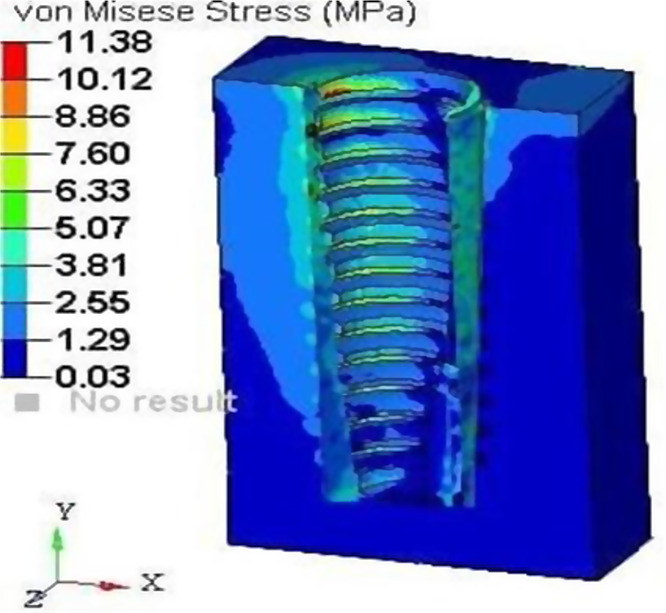
Stress on the bone of SS of thickness 2.0 mm

The implant was subjected to a vertical loading torque of 40 Ncm, whereas the root structure and bone were loaded with a horizontal force of 20 N. The analysis depicted peak stress on the root structure, measuring 2.0 mm in thickness, at 28.74 MPa (Fig. 22), and on the bone at 11.38 MPa (Fig. 23), with principal strain values of 1.55E-02 and 8.31E-03, respectively.

**Fig. 24:**
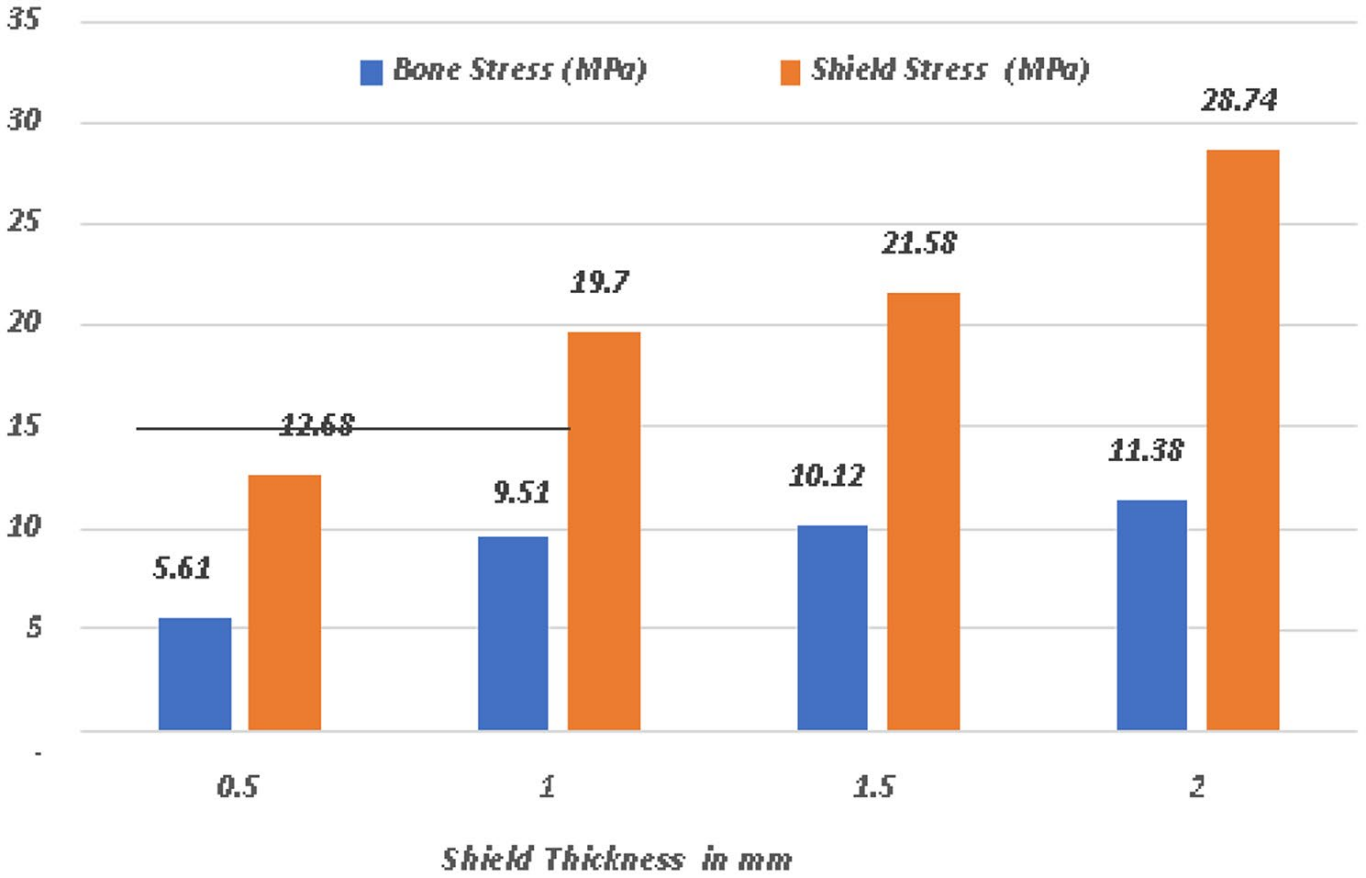
Comparative analysis of von Misses stress in shield and bone

**Fig. 25:**
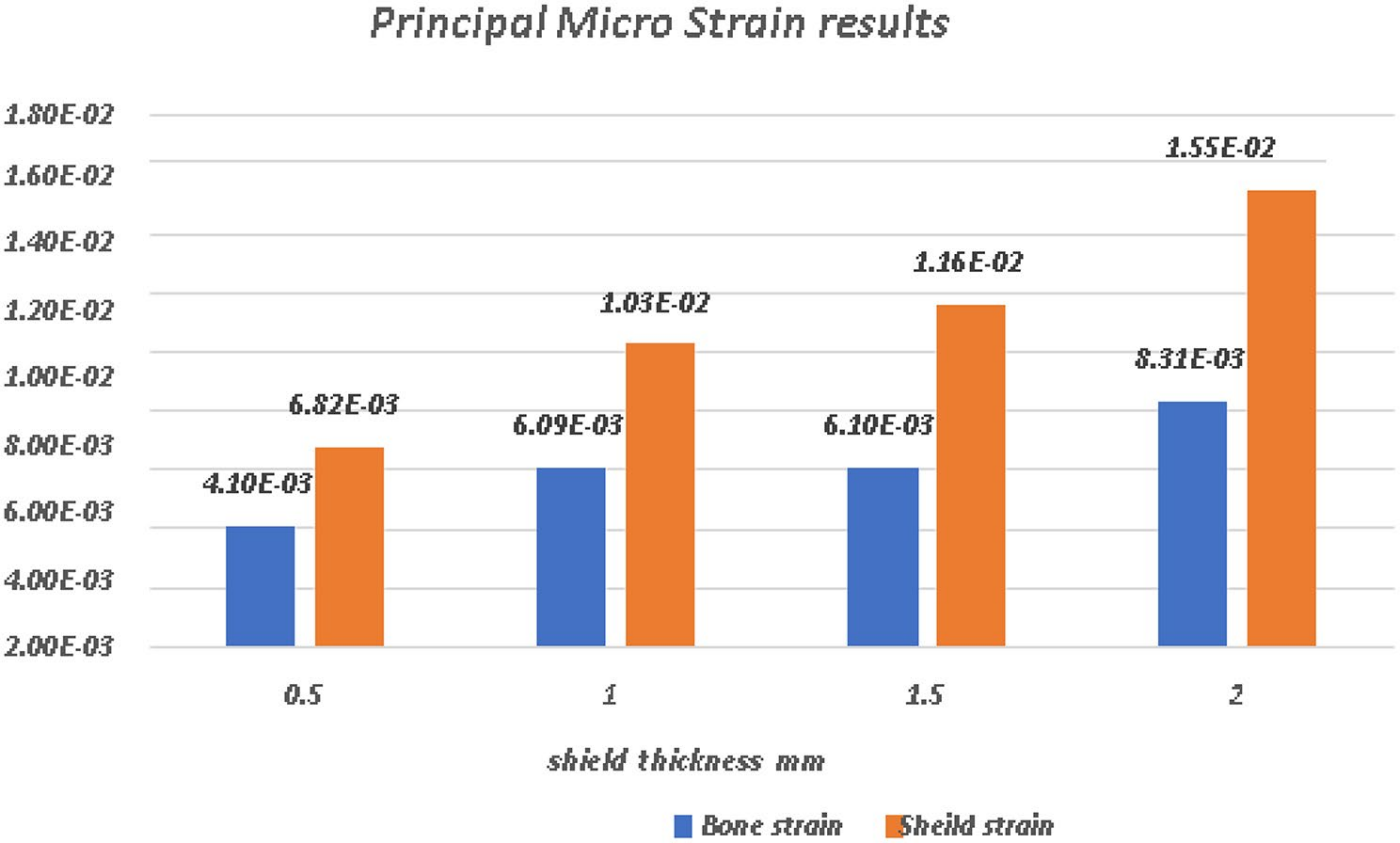
Comparative analysis of principal micro-strain in shield and Bone

**Fig. 26:**
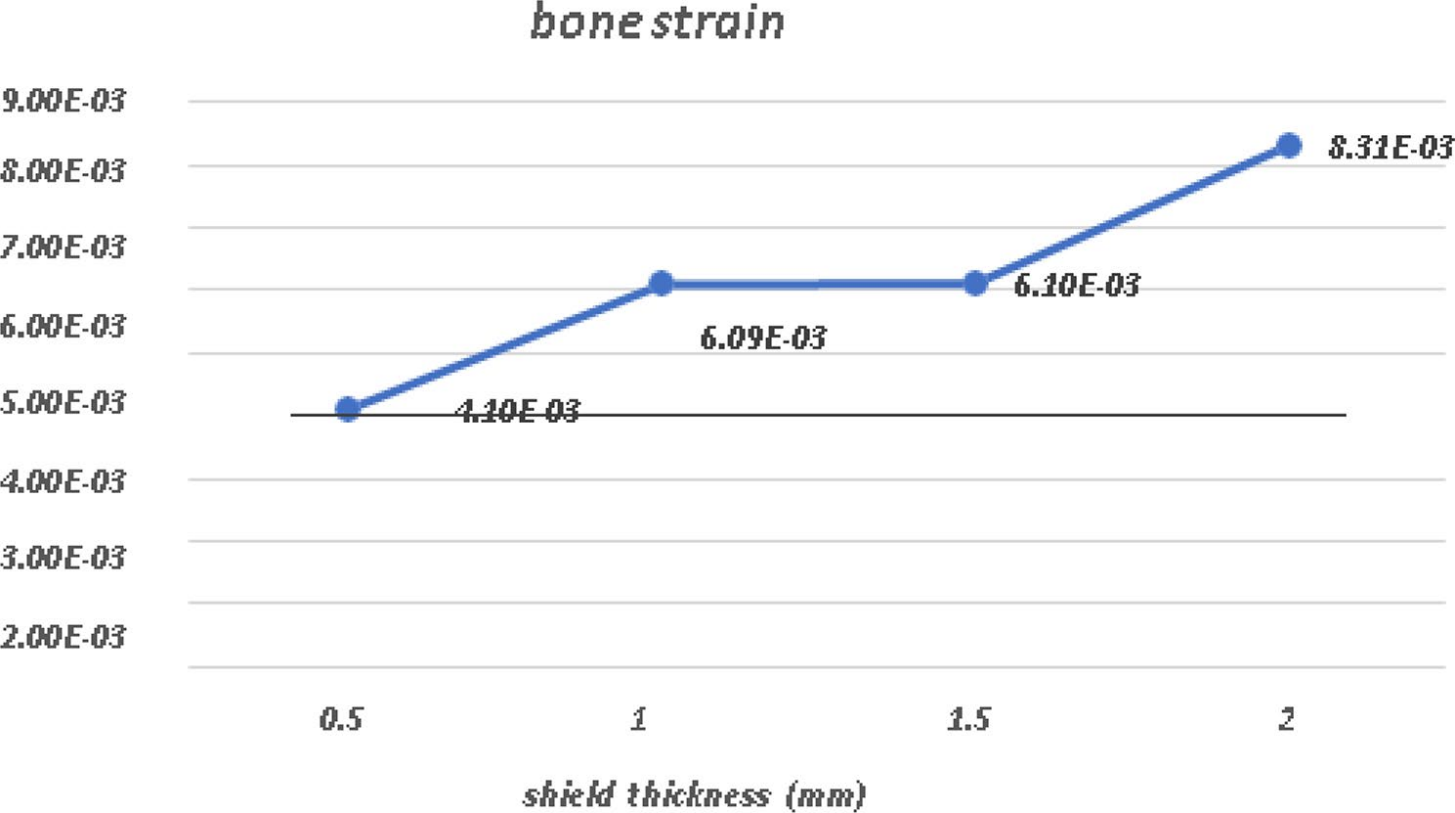
Principal micro-strain on Bone

**Fig. 27:**
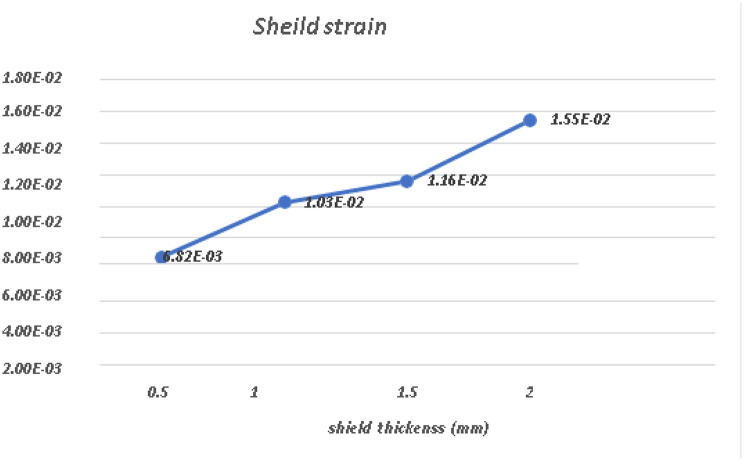
Principal micro-strain on the shield

A comparative analysis of the von Mises stress and principal microstrain was conducted. The various readings were tabulated in Table 3 for von Misses stress generation on different shield thicknesses, and a comparative analysis of stress generation for different shield thicknesses is depicted in Fig. 24. The readings obtained for the principal microstrains are listed in Table 4. A comparison between the strain values for different shield thicknesses is plotted in Fig. 25. A separate analysis of the strain generation on the bone is shown in Fig. 26. The strain generation on the shield is shown in Fig. 27. Based on the findings of this investigation, it can be shown that the use of the socket shield technique and immediate implant insertion techniques resulted in heightened stress and strain on both the bone and shield. Furthermore, the stress and strain increased proportionally with the thickness of the shield.

**Table 3:** Von Misses stress on shield and bone in various socket shield thicknesses

Shield thickness in mm	Bone stress (Mpa)	Shield stress (Mpa)
0.5	5.61	12.68
1.0	9.51	19.07
1.5	10.12	21.58
2.0	11.38	28.74

**Table 4:** Principal micro-strain on shield and bone in various socket shield thicknesses

Shield thickness in mm	Bone strain (mm/mm)	Shield strain (mm/mm)
0.5	4.10E-03	6.82E-03
1.0	6.09E-03	1.03E-02
1.5	6.10E-03	1.16E-02
2.0	8.31E-03	1.55E-02

### Inference

When the thickness of the shield increases during the socket shield operation for immediate implant insertion, there is a concomitant increase in the tension exerted on both the root fragment and surrounding bone. When the thickness of the shield in the socket shield technique increases immediately following dental implantation, there is a concurrent increase in the primary microstrain.

## Discussion

The objective of this study was to employ finite element analysis to examine the distribution of stress and ascertain the optimal thickness of labial root fragments in socket shield operations for rapid dental implant insertion.

Implant placement immediately after tooth extraction is a well-established treatment approach. However, the success of implant treatment is no longer solely measured by its survival; it also relies on achieving aesthetic and functional success in the final prosthetic treatment. This success is heavily influenced by the optimal positioning of the implant, which in turn is influenced by its vertical and horizontal placement and is dependent on the dimensions of the alveolar ridge. Despite the utilization of various treatment methods, such as graft materials and barrier membranes, to mitigate the negative effects of tissue remodeling following extraction, complete preservation of ridge dimensions remains unattained.

The concept of the socket shield technique emerged from early ideas dating back to 1950, which suggested that retaining the root along with the periodontal ligament and fibers could limit tissue alterations following extraction, thereby minimizing the physiological remodeling of the extraction socket.

Schwimmer et al. [9] presented the initial histological findings in humans, suggesting the possibility of complete bone filling between the osseointegrated implant surface and root dentin in an unplanned dental implant placed using the socket shield technique (SST). Similarly, Mitsias et al. [10] provided additional human histological evidence of immediate implant placement in the anterior maxilla using the root membrane technique (RMT). In a case study, a patient who had an implant for five years experienced an accident that required surgical removal of the fixture. Histological evaluation of the implants revealed a healthy periodontal ligament with increased bone-to-implant contact (76.2%) and no resorption of the buccal bone plate. Histological evidence from the socket shield technique confirmed that the attachment of the remaining root fragment through the physiological periodontal ligament did not elicit an inflammatory response. Moreover, the bone crest exhibited no signs of active remodeling, and the coronal soft tissue demonstrated a physiological junctional epithelium without any inflammatory response.

Siormpas et al. [11] conducted a comprehensive study to examine the long-term effects of the socket shield technique (SST) on dental implants. In this study, the thickness of the buccal portion of the root was reduced to 1.5 mm, and the implants were followed up for ten years. Of the total number of implants, only five failed, with two failures attributed to osseointegration issues and three due to peri-implantitis. The overall survival rate was 98%. Additionally, only three cases of complications related to infection of the root membrane, which were easily treated, have been reported. These findings support the assertion that SST is a safe and reliable technique associated with minimal biological complications.

While previous studies have suggested that the socket shield technique can preserve bucco-facial tissues, it is important to note that absolute preservation is yet to be documented. In 2010, Baumer et al. [12] examined volumetric changes in the alveolar ridge and reported a mean loss of 0.88 mm in the labial area, with a maximum loss of 1.67 mm and a minimum loss of 0.15 mm. Another study by Chen et al. [13] in 2013 reported a bone resorption of approximately 0.72 mm. Although these studies provided valuable insights into post-extraction ridge alterations on the buccal side, they did not specify the exact dimensional changes before and after SST. However, a randomized trial conducted by Bramanti et al. [14] compared SST with the conventional technique for post-extraction implants over a 3-year follow-up period. The results showed that SST was safe and offered superior aesthetic outcomes compared to the conventional technique.

The finite element method (FEM) is a numerical technique used to analyze and evaluate the stresses and deformations in structures with different geometries. This method involves discretizing the structure into finite elements that are interconnected through nodes. In the field of implantology, FEA has been utilized to examine stress patterns in various components of implants, as well as in the surrounding peri-implant bone.

Several FEA studies have postulated that the stress distribution on the peri-implant bone is impacted by a range of elements. These factors encompass the characteristics of the implant, such as the number, diameter, and length, as well as the thread profile. Additionally, the material properties of the implant components and the quality and quantity of the surrounding bone are also believed to influence stress distribution. Various stress analyses were utilized in these studies to evaluate the mechanical stress endured by the peri-implant bone. These analyses included the von Mises stress, maximum and lowest main stresses, and maximum shear stress.

Among these stress parameters, the von Mises stress is a commonly used scalar-valued stress invariant that provides insights into the yielding and failure behavior of different materials. The maximum principal stress is suitable for evaluating the tensile stress, whereas the minimum principal stress indicates compressive stress. Considering that bone exhibits both ductile and brittle properties, the use of principal stress is appropriate for such analyses.

In a relevant FEA study, Demenko et al. [15] recommended careful selection of implant size, with emphasis placed on its load-bearing capacity.

Calvo-Guirado et al. [16] conducted a randomized controlled experiment to examine the influence of root fragment length and position on the breadth of buccal peri-implant bone. The study encompassed a sample of six dogs and a total of 48 implants that were inserted with the SST. The results of this study indicate that the coronal radicular fragment, which is connected to the buccal bone plate by the physiological periodontal ligament (PDL), had a lower degree of crestal bone resorption than the middle and entire root groups. The outcomes were affected by the presence of extended root fragments surrounding the implant, as evidenced by the observation that samples with root fragments exceeding 2 mm exhibited heightened levels of bone remodeling and migration of bone towards the apex.

In a separate randomized controlled trial by Tan et al. [17] involving four dogs and 16 implants with remaining root segments, the researchers aimed to determine the resorption of the buccal bone. The study revealed that the height of the root segment had minimal effects on bone absorption, whereas root segments measuring 0.5-1.5 mm demonstrated reduced bone resorption. Thinner root sections were found to result in fewer adverse reactions, such as vibration and heat generation, during the preparation of implant beds while also maintaining a healthy PDL and assisting in soft tissue preservation. Additionally, the SST group exhibited shallower pocket probing depths than the control group.

Few studies have been conducted to evaluate different thicknesses and stress distributions on the residual root structure using the socket shield technique. Therefore, this study aimed to determine the stress distribution on the residual root structure using the socket shield technique and immediate implant placement using finite element analysis.

In this study, a torque of 40Ncm vertically and a force of 20 N were applied horizontally on the implant. A force of 20 N was derived because the torque was a product of the force multiplied by the radius. As the torque and radius of the implant are known to be 40 Ncm and 2 mm, respectively, the resultant force is 20 N, which is primarily applied to the inner aspect of the shield.

After the application of force on model A, that is, the socket shield with a thickness of 0.5 mm, the von Misses stress generated on the shield was 12.68Mpa, and on bone, it was 5.61Mpa. The principal microstrains were 6.82E-03 and 4.10E-03 for the shield and bone, respectively. Similarly, when the same amount of torque and force were applied to model B with a shield thickness of 1 mm, the von Mises stress generated was 19.07Mpa on the shield and 9.51Mpa on the bone. The principal strains generated in this model were 1.03E-02 and 6.09E-03 for the shield and bone, respectively. When the same conditions were applied to model C with shield thicknesses of 1.5, the maximum stress on the root structure was 21.58 Mpa and that on the bone was 10.12 Mpa, and the principal strains were 1.16E-02 and 6.10E-03, respectively. In model D that is with socket shield thickness of 2 mm, the stress and strain values were maximum; that is, the von Misses stress on the shield was 28.74 Mpa on the bone of 11.38 Mpa and principal strain of 1.55E-02 and 8.31E-03 respectively.

According to the systematic review conducted by Akanksha Mourya [18] and studies conducted by Tribst et al. [4], JW Stanford [19], Xi Ding et al. [20], and Ikumi et al. [21], the results obtained for the stress generation suggest that the use of shield thickness of 0.5 to 1.5 mm does not cause any detrimental stress generation on the residual root fragment, however considering the implant sizes and the diameter of the socket it is safe to consider the maximum dimension of the root fragment of 1.5 mm for the socket the technique. Thus, the analysis supports the use of root fragments of thickness ranging from 0.5 to 1.5 mm, as reported in previous studies. Stress generation on the root fragment increased with increasing root fragment thickness. Currently, there is a lack of literature discussing the biomechanical response and stress generation specific to socket shields of varying thicknesses. Consequently, this study aims to fill this gap and serves as a pioneer in exploring this particular aspect of the socket shield technique.

The findings of this study provide valuable insights for clinicians when considering the use of the socket shield technique. The study suggests that a shield thickness ranging from 0.5 to 1.5 mm can be employed without inducing excessive stress on the surrounding bone. Therefore, it is recommended to implement the same postoperative procedures utilized in conventional techniques to achieve long-term success and prevent undesired bone remodeling.

The socket shield approach is based on the maintenance of periodontal ligament nourishment and establishes a robust theoretical basis. Therefore, the aesthetic improvements reported in this methodology, which are ascribed to the preservation of bone tissue, do not appear to be associated with alterations in the stress distribution within the peri-implant tissues.

In this study, an implant was simulated within a bone model by incorporating socket shields of various thicknesses. A static load of 20 N was applied using a mathematical formula based on a known torque value of 40 N and the radius of the implant (2 mm). It is important to note that this study only examined the effects of static loading and used single-diameter implants. Therefore, further research is necessary to investigate the biomechanical behavior of the socket shield technique under dynamic loading conditions, considering different implant diameters, to assess frictional resistance and stress generation on the shield.

It is important to acknowledge that finite element analysis constitutes a theoretical methodology that possesses inherent limits in its ability to comprehensively encompass all variables. The present study did not consider variables such as temperature, fluctuations in pH, and diverse loading conditions. Furthermore, it was assumed in the research that the simulated materials utilized were both isotropic and devoid of defects. However, it is important to acknowledge that these assumptions may not fully mirror the circumstances observed in real-world clinical settings.

## Conclusions

Based on the findings of the study, the following conclusions can be drawn

1. Increasing the shield thickness (from 0.5 to 2 mm) in the socket shield process during implant placement leads to higher stress production and also primary micro-strain increases on the root fragment and bone.
2. The use of a shield thickness of 0.5 to 2 mm does not cause any detrimental stress generation on the residual root fragment; however, considering the implant sizes and the diameter of the socket, it is safe to consider the maximum dimension of the root fragment of 1.5 mm for the socket shield technique.

## Data Availability

The data supporting this study’s findings are available from the corresponding author upon reasonable request.

## Abbreviations

FEA: Finite Element Analysis
PDEs: Partial Differential Equations
DOF: Degrees of Freedom
SST: Socket Shield Technique
RMT: Root Membrane Technique
FEM: Finite Element Method
PDL: Periodontal Ligament
